# Distribution Patterns and Determinants of Invasive Alien Plants in China

**DOI:** 10.3390/plants12122341

**Published:** 2023-06-16

**Authors:** Jing Chen, Yanjing Zhang, Wei Liu, Chenbin Wang, Fangzhou Ma, Haigen Xu

**Affiliations:** Nanjing Institute of Environmental Sciences, Ministry of Ecology and Environment of China, Nanjing 210042, China; chenjing_0314@163.com (J.C.);

**Keywords:** first records, similarity coefficients, synthetic score, environmental factors, anthropogenic factors, congruence

## Abstract

In recent years, invasive alien plants (IAPs) have caused serious ecological disasters and economic losses in China. This study combined three IAP species richness-related indices (species richness of IAPs, first records of IAPs, and the relative species richness of IAPs), as well as indices reflecting distribution and dispersal patterns (average similarity coefficient of IAPs) and invasiveness (average risk score of IAPs), to conduct an integrated regional-invasion risk assessment based on the principal component analysis (PCA) method. Partial least-squares (PLS) regression was conducted to explore the explanatory power of 12 environmental and anthropogenic factors on different invasion indices. The results indicated that coastal provinces and Yunnan had high IAP introduction risk, as well as high synthetic-risk scores. The dispersal of IAPs in mid-latitude provinces should be particularly prevented. For species richness of IAPs, more environmental factors with variable importance for the project (VIP) values higher than 1 were retained in the optimal model, reflecting the importance of environmental filtering on IAPs. Visitors were the most important predictor for first records of IAPs. Compared to species richness (*R*^2^ = 79.5%), first records were difficult to predict (*R*^2^ = 60.4%) and were influenced by anthropogenic factors. There was spatial distribution congruence of various families of IAPs. Generally, the correlations of the residuals of species richness were still significant, with 0.421 (*p* < 0.05) as the lowest Pearson correlation coefficient, which indicated that external factors could not fully explain the spatial distribution congruence. These findings could enrich the relevant research on IAP invasion mechanisms and provide suggestions for regional IAP detection and response.

## 1. Introduction

Invasive alien plants (IAPs) have negative impacts on both the economy and ecology [1]. Almost all types of indigenous plant communities, including protected areas, are threatened by the invasion of non-native plant species [1,2]. It has been estimated that in New Zealand alone, the annual losses caused by IAPs exceed USD 1 billion [3]. Biological invasions in China have resulted in an estimated economic loss of over USD 14.5 billion per year [4]. In recent years, global climate change and ongoing economic development have accelerated the colonization and spread of IAPs [5,6]. Over the past 200 years, the annual growth rate of recorded global alien species introductions has continued to increase, with the first records reported from 1970 to 2014 alone, accounting for 37% of all records [7]. In addition, during the period from 2000 to 2005, on average, a quarter of the first records of alien species were new and were recognized as “emerging alien species” [8]. It has been pointed out that past efforts to control or mitigate biological invasion have not achieved significant results. As highlighted in the mid-term analysis of the Strategic Plan for Biodiversity 2011–2020 under the Convention on Biological Diversity, current efforts remain insufficient to reduce the accumulation of alien species to a large extent [9].

It was recorded that the introduction of alien plants in China significantly increased around the Ming Dynasty (1368–1644) [10]. In recent years, the cumulative number of IAPs has continued to increase [11,12,13]. China has a vast land area, spanning five climate zones from tropical to cold temperate and covering multiple biodiversity hotspots [14]. With international trade, tourism, and other interactions being increasingly frequent in China, species exchanges between regions are constantly breaking through geographical barriers [15]. After the introduction of exotic plants into new sites, the success of an invasion depends on factors such as propagule pressure, the invasiveness of plant species, and the invasibility of ecosystems [1,16]. The importance of environmental factors, such as temperature and precipitation, is in the susceptibility of IAPs to environmental conditions of introduction regions [17,18,19,20,21,22,23,24]. Anthropogenic factors, such as population, trade, transportation, and tourism development, could directly increase the frequency of the introduction of alien species and propagule pressure (for example, commercial forestry and ornamental horticulture) [17,23,24,25,26], and economic activities could also result in an increase in human disturbance and eutrophication and indirectly promote the colonization or dispersal of IAPs [1,27]. For example, human-mediated activities have led to the fragmentation of the habitat on which indigenous plants depend, resulting in the occurrence of suitable ecological niches for IAPs [28]. The species composition, spatial distribution, and relationship with the environment and anthropogenic activities of IAPs have always been a hot topic in the study of biological invasions [1,29,30]. Previous studies have shown that the species richness of IAPs in China decreases from the southeast coast to the northwest inland, with the southern region being the most severely affected by IAPs [31]. The distribution patterns of IAPs are related to natural and socio-economic factors, and factors such as temperature, precipitation, potential evapotranspiration (PET), and gross domestic product (GDP) have great explanatory power [31,32,33].

Previous invasion risk assessments mainly focused on species [34,35]. For example, Ma and Li ranked IAPs based on their invasion characteristics [36], while Zhou et al. rated species based on their distribution and dispersal characteristics [37]. In addition, studies have evaluated the invasion risks for regions, with species richness being the most studied indicator, and other derived indices, including the species richness percentage, species richness density, and species richness weighted by impact and economic indices [32,33]. Several studies have combined these indices with some of characteristics of IAPs [33,38]. Firstly, in this study, three IAP species richness-related indices (species richness, first records, and relative invasive species richness of IAPs) were combined with indices reflecting distribution and dispersal patterns (the average similarity coefficient of IAPs) and invasiveness (the average risk score of IAPs) to conduct a more integrated regional-invasion risk assessment. Then, this study also explored the explanatory power of environmental and anthropogenic factors on various invasion indices. Finally, because the species richness of IAPs of different families was highly correlated, this study further explored the contributions of environmental and anthropogenic factors to this congruence. This study aims to enrich the relevant research on IAP invasion mechanisms and provide some suggestions for early detection and spread blocking, as well as provide some basic information for setting management intensity and aspects in different regions.

## 2. Results

### 2.1. Composition of IAPs in China

The list included a total of 368 IAPs. At the family level, the IAPs belonged to 67 families, of which four families included 20 or more species: Compositae (72 species); Leguminosae (51 species); Poaceae (45 species); and Amaranthaceae (20 species). There were four families with 10 to 19 species: Solanaceae (19 species); Euphorbiaceae (13 species); Onagraceae (13 species); Convolvulaceae (13 species); and Brassicaceae (10 species). There were 35 families containing only one species, seven families containing two species, six families containing three species, five families containing four species, three families containing five species (Verbenaceae, Apiaceae, and Cactaceae), and two families containing six species (Myrtaceae and Scrophulariaceae). IAPs in the Compositae, Amaranthaceae, Poaceae, and Solanaceae families were mainly annual or biennial, while in the Leguminosae family, IAPs were mainly perennials, shrubs, or trees. The chi-squared (*χ*^2^) test results indicated that there were differences in the life forms of IAPs from different families (annual or biennial herbs vs. perennials or shrubs and trees) (*χ*^2^ = 24.694, *p* < 0.001).

### 2.2. IAP Distribution Patterns in China

#### 2.2.1. IAP Species Richness

**Figure 1:**
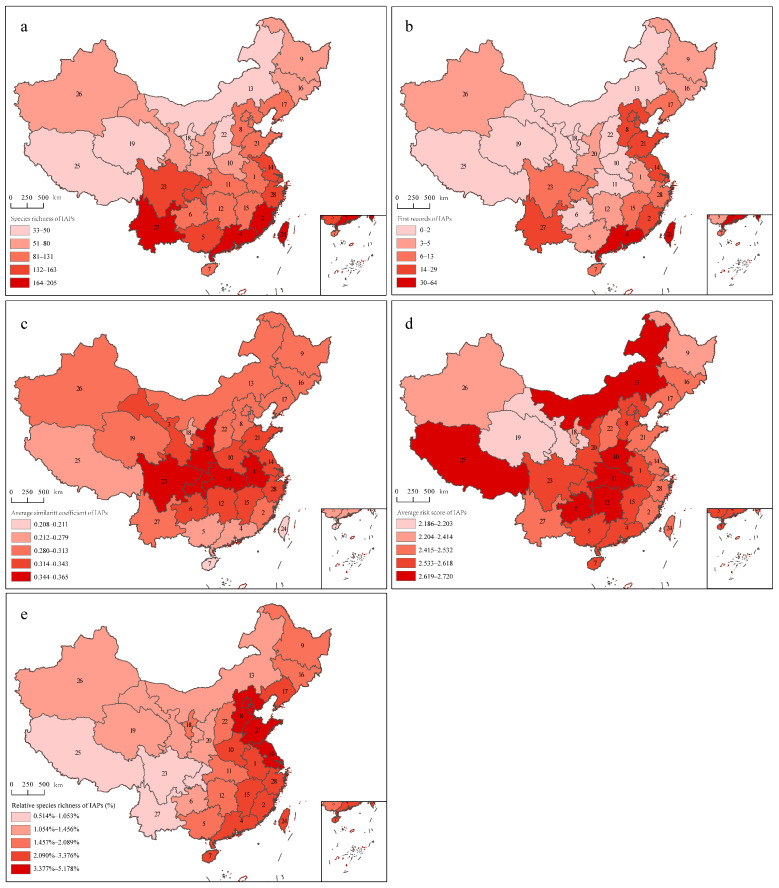
Maps of five invasion indices in China. It is noted that (**a**–**e**) represent the species richness of IAPs, the first records of IAPs, the average similarity coefficient of IAPs, the average risk score of IAPs, and the relative species richness of IAPs, respectively. The numbers in the maps 1–28 represent 28 provinces in China: 1, Anhui; 2, Fujian; 3, Gansu; 4, Guangdong (including Hong Kong and Macao); 5, Guangxi; 6, Guizhou; 7, Hainan; 8, Hebei (including Beijing and Tianjin); 9, Heilongjiang; 10, Henan; 11, Hubei; 12, Hunan; 13, Inner Mongolia; 14, Jiangsu (including Shanghai); 15, Jiangxi; 16, Jilin; 17, Liaoning; 18, Ningxia; 19, Qinghai; 20, Shaanxi; 21, Shandong; 22, Shanxi; 23, Sichuan (including Chongqing); 24, Taiwan; 25, Tibet; 26, Xinjiang; 27, Yunnan; and 28, Zhejiang.

**Figure 2:**
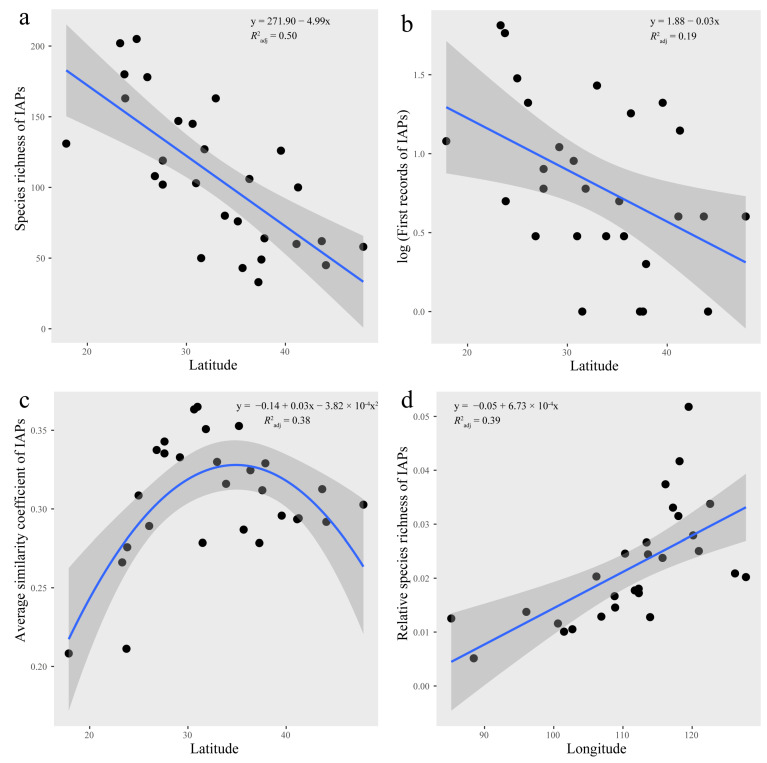
Distribution pattern of four invasion indices along latitude or longitude. Shaded area around fitted trend line indicates 95% confidence interval. It is noted that (**a**–**d**) represent the relationship between species richness of IAPs and latitude, the relationship between log-transformed first records of IAPs and latitude, the relationship between average similarity coefficient of IAPs and latitude, and the relationship between relative species richness of IAPs and longitude, respectively.

The provinces with the highest IAP species richness were Yunnan and Guangdong, with 205 and 202 species, respectively, followed by Taiwan, Fujian, Guangxi, and Jiangsu, with IAP species richness of above 150 species; Ningxia had the lowest value, followed by Qinghai, Inner Mongolia, Shanxi, and Tibet, with IAP species richness of no more than 50 species (Figure 1a). The ordinal least-squares (OLS) regression results showed that IAP species richness significantly increased with decreasing latitude; that is, it increased from north to south (Figure 2a).

#### 2.2.2. First Records of IAPs

The first records of IAPs showed that 64 IAPs first appeared in Guangdong, 57 species first appeared in Taiwan, and 29 species first appeared in Yunnan Province. In addition, there were also many first records in provinces including Jiangsu, Hebei, Fujian, and Shandong, while there were no first records in Ningxia, Tibet, Shanxi, and Inner Mongolia (Figure 1b). The OLS regression results showed that, similar to the IAP species richness, the first records of IAPs significantly increased with decreasing latitude; that is, the first records increased from the north to the south (Figure 2b).

#### 2.2.3. Average Similarity Coefficient of IAPs

**Figure 3:**
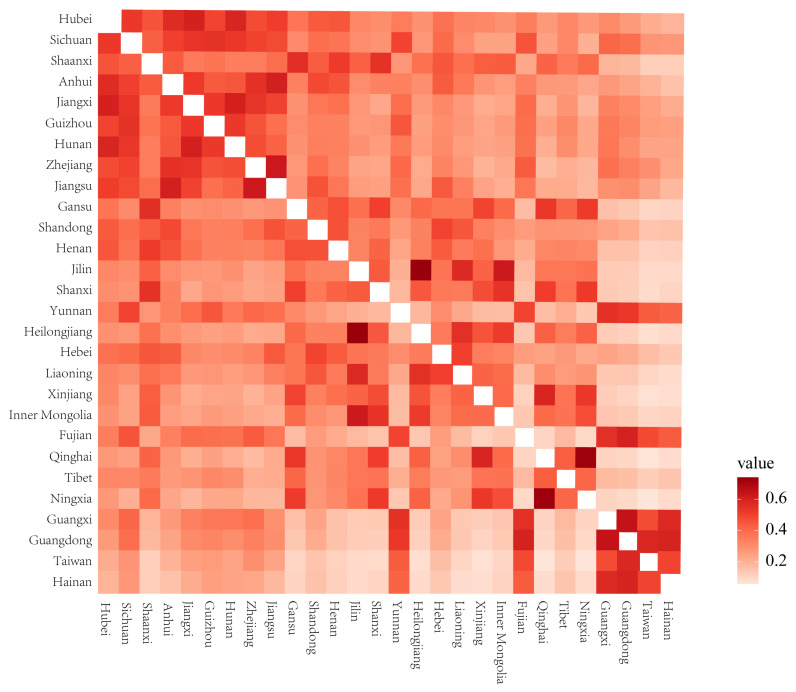
Jaccard’s similarity coefficients of IAP species composition between different provinces.

In general, the provinces with high similarity coefficients were often geographically adjacent provinces. Jilin and Heilongjiang had the highest similarity coefficient of 0.74, followed by Gansu and Qinghai, with a similarity coefficient of 0.73. The similarity coefficients of Guangdong and Guangxi, Jiangxi and Hubei, Jiangxi and Hunan, Fujian and Guangdong, Jiangsu and Zhejiang, Jiangsu and Anhui, and Inner Mongolia and Jilin were above 0.6. The similarity coefficients between Tibet and other provinces were not high, which might be due to its unique plateau climate. Henan only had one similarity coefficient higher than 0.5, which was with Shaanxi. Hebei, Xinjiang, and Taiwan had similarity coefficients higher than 0.5 with two provinces (Figure 3). According to the average similarity coefficients of IAPs, Hainan had the lowest, followed by Taiwan; Hubei had the highest, followed by provinces including Sichuan, Shaanxi, and Anhui (Figure 1c). As the latitude increased, there was a trend in which the similarity coefficients first increased and then decreased, with the average similarity coefficients lower in the southern and northern provinces, especially in the southern provinces, and the average similarity coefficients of provinces higher in the middle latitude (Figure 2c).

#### 2.2.4. Average Risk Score of IAPs

The distribution of invasion risk scores varied among different families. Compared with other families, Compositae had a high proportion of malignant IAPs, with 18 malignant IAPs, including Ageratina adenophora, Solidago canadensis, Mikania micrantha, and Flaveria bidentis, and the next was Amaranthaceae. The average risk score of Amaranthaceae was the highest (2.90 ± 1.1447), followed by Compositae (2.82 ± 1.604), Solanaceae (2.53 ± 1.349), Poaceae (2.27 ± 1.355), other families (2.13 ± 1.280), and Leguminosae (1.75 ± 1.129). The Kruskal–Wallis test results showed significant differences in the risk scores of IAPs among different families (*χ*^2^ = 22.86, *p* < 0.001). A Bonferroni post-hoc test found that there were significant differences in the risk scores between Compositae and Leguminosae (adjusted *p* < 0.001), Compositae and other families (adjusted *p* = 0.03), and Leguminosae and Amaranthaceae (adjusted *p* = 0.02). The average risk score of IAPs in Tibet was the highest at 2.72, followed by Henan at 2.70, and Qinghai had the lowest at 2.19 (Figure 1d). However, the average risk score of IAPs in each province showed no significant trends in either latitude or longitude.

#### 2.2.5. Relative Species Richness of IAPs

The IAPs of the Onagraceae and Solanaceae families accounted for the largest proportion of their respective native plants, while for Compositae, Leguminosae, and Poaceae, although these families contained a large number of IAPs, they did not account for a significant proportion of native plants. The relative species richness of IAPs for each province showed that Jiangsu Province had the highest at 5.2%, followed by Shandong Province at 4.2%; Tibet had the lowest at only 0.5%, with Yunnan slightly higher at 1.0% (Figure 1e). The relative species richness of IAPs significantly increased with the increase in longitude, meaning that the relative species richness of IAPs increased significantly from the western provinces to the eastern coastal provinces (Figure 2d).

#### 2.2.6. Synthetic Score of IAP Invasion Risk

**Figure 4:**
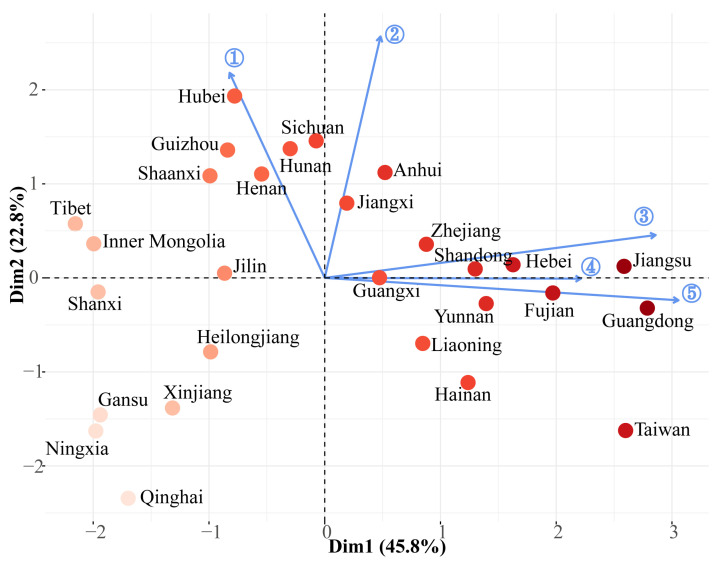
Results of synthetic scores of five invasion indices based on principal component analysis (PCA) method. Arrow ①, ②, ③, ④, and ⑤ represent the average similarity coefficient of IAPs, the average risk score of IAPs, the species richness of IAPs, the relative species richness of IAPs, and the first records of IAPs.

**Figure 5:**
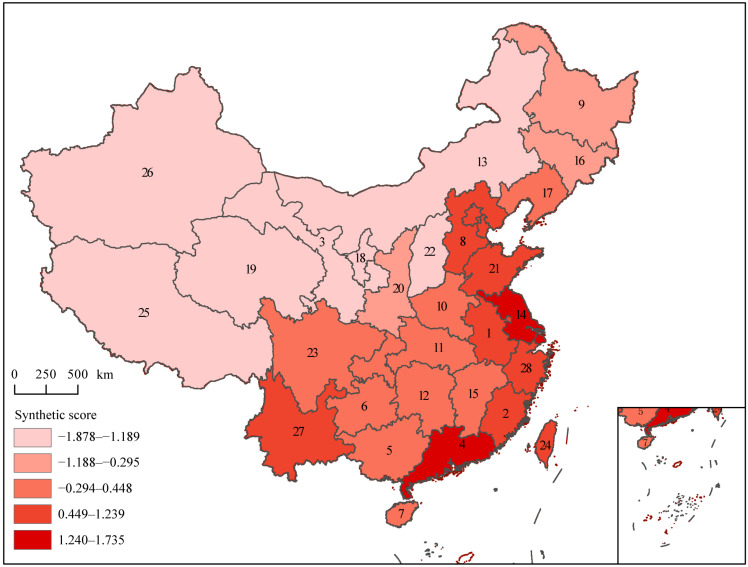
Map of the synthetic scores of five invasion indices based on PCA. Numbers 1–28 in the map represent 28 provinces in China: 1, Anhui; 2, Fujian; 3, Gansu; 4, Guangdong (including Hong Kong and Macao); 5, Guangxi; 6, Guizhou; 7, Hainan; 8, Hebei (including Beijing and Tianjin); 9, Heilongjiang; 10, Henan; 11, Hubei; 12, Hunan; 13, Inner Mongolia; 14, Jiangsu (including Shanghai); 15, Jiangxi; 16, Jilin; 17, Liaoning; 18, Ningxia; 19, Qinghai; 20, Shaanxi; 21, Shandong; 22, Shanxi; 23, Sichuan (including Chongqing); 24, Taiwan; 25, Tibet; 26, Xinjiang; 27, Yunnan; and 28, Zhejiang.

**Figure 6:**
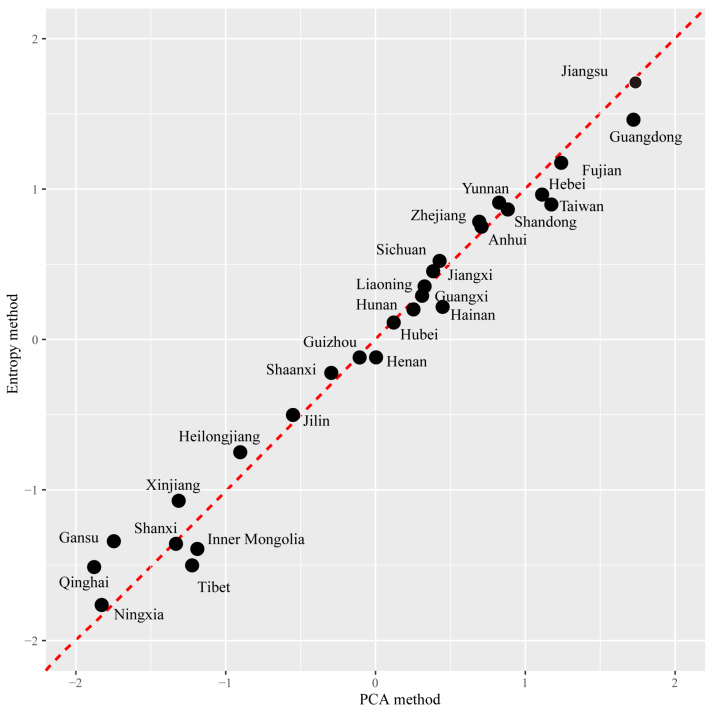
Comparison of PCA scores and entropy method scores.

Principal component analysis (PCA) of five invasion indices showed that the first two components explained 68.6% of the variation (Figure 4). The risk score and similarity coefficient had greater correlations with the second component, while the other three indices had greater correlations with the first component (Figure 4). Jiangsu Province had the highest synthetic score, followed by Guangdong, Fujian, Taiwan, Hebei, Shandong, Yunnan, Anhui, and Zhejiang (Figure 4 and Figure 5). Qinghai had the lowest synthetic score, followed by provinces Ningxia, Gansu, Shanxi, Xinjiang, Tibet, and Inner Mongolia (Figure 4 and Figure 5). The ranking results based on the entropy method were highly similar to the PCA results, confirming the credibility of the PCA results (Figure 6).

### 2.3. Impacts of Multiple Factors on the Spatial Distribution of IAPs

**Figure 7:**
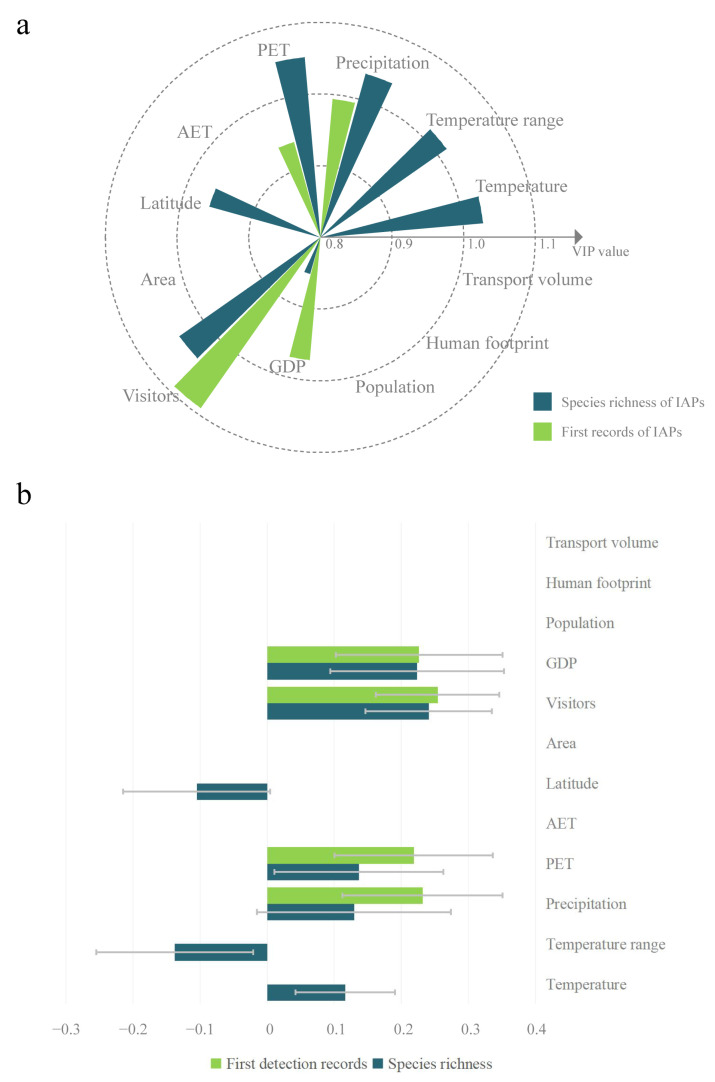
Results of PLS regression analysis of invasion indices and environmental and anthropogenic factors. Figure (**a**) shows the results of the variable importance for the project (VIP) values. Figure (**b**) shows the results of regression coefficients. Temperature range represents the temperature annual range; PET represents the potential evapotranspiration; AET represents the actual evapotranspiration; and visitors represent the number of visitors. The VIP values of four predictors (area, population, transport volume, and human footprint) were empty because they were removed from both of the optimal models.

The partial least-squares (PLS) regression results for species richness showed that the model extracted two components, which accounted for 79.5% of the variation. The *Q*^2^_cum_ of the model was 0.763, indicating that the PLS regression model was well fitted and had high robustness. In the optimal PLS model, a total of seven explanatory variables were retained, and the variable importance for the project (VIP) values of five variables were greater than 1, indicating their high importance. The PET was the highest, followed by the number of visitors (hereinafter referred to as visitors), precipitation, temperature, and temperature annual range (hereinafter referred to as temperature range) (Figure 7a). The regression coefficients of the latitude and temperature range were negative, while those of the other five variables were positive (temperature, precipitation, PET, visitors, and GDP) (Figure 7b).

The PLS regression results of the first records showed that the model extracted a total of one component and explained 60.4% of the variation. The *Q*^2^ of the model was 0.573. The fitting ability and robustness of the PLS regression model were lower than that of species richness. In the optimal PLS model, a total of four explanatory variables were retained (visitors, precipitation, GDP, and PET), and only the VIP value of visitors was greater than 1, indicating its higher importance (Figure 7a). The regression coefficients of all retained variables were significantly positive (Figure 7b). Overall, compared with the IAP species richness, the first records of IAPs were difficult to predict, and anthropogenic factors had great impacts (such as visitors).

### 2.4. Impacts of Multiple Factors on the Spatial Distribution of Different Families of IAPs

**Figure 8:**
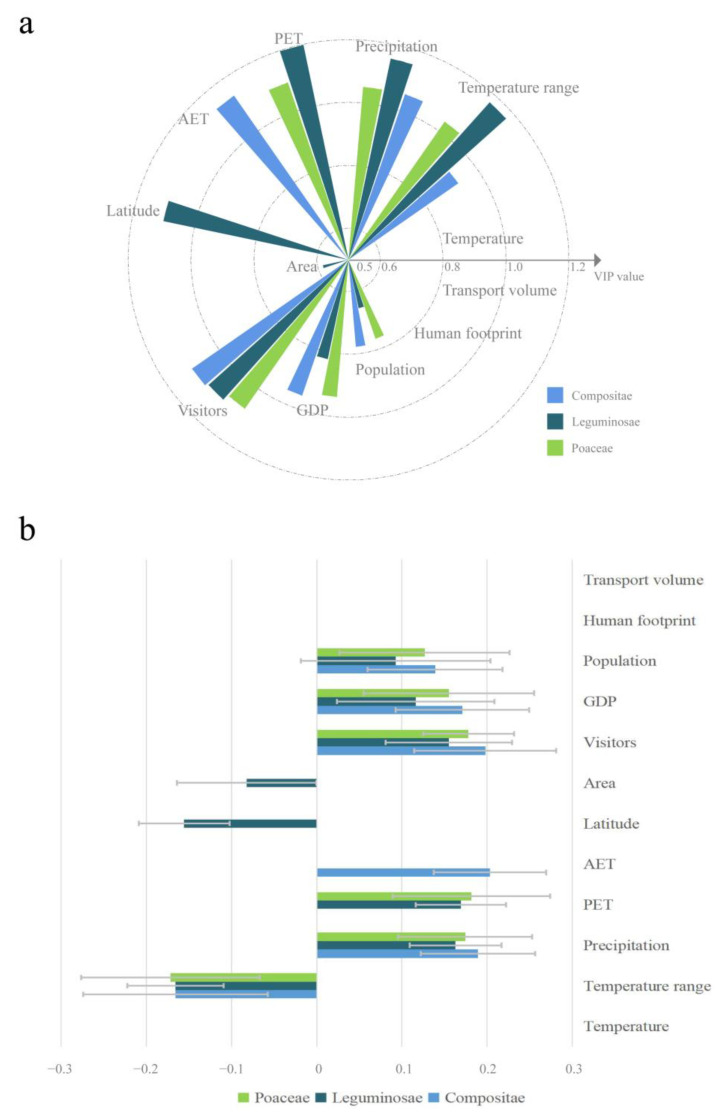
Results of PLS regression analysis of IAP species richness of Compositae, Leguminosae, and Poaceae families and environmental and anthropogenic factors. Figure (**a**) shows the results of VIP values. Figure (**b**) shows the results of regression coefficients. Temperature range represents the temperature annual range; PET represents the potential evapotranspiration; AET represents the actual evapotranspiration; and visitors represent the number of visitors. The VIP values of three predictors (transport volume, human footprint, and temperature) were empty because they were removed from all of the optimal models.

The PLS regression results for Compositae, Leguminosae, and Poaceae showed that one component was extracted for each family that explained 75.0%, 75.5%, and 61.3% of the variation in the explanatory variables, respectively. For Compositae, a total of six explanatory variables were retained in the optimal PLS model, and the VIP values of three variables were greater than 1 (AET, visitors, and precipitation), indicating their high importance (Figure 8a). For Leguminosae, eight explanatory variables were retained, and the VIP values of five variables were greater than 1 (PET, temperature range, precipitation, latitude, and visitors) (Figure 8a). For Poaceae, six explanatory variables were retained, and the VIP values of four variables were greater than 1 (PET, visitors, precipitation, and temperature range) (Figure 8a). The standardized regression coefficients of all retained predictors of Asteraceae, Leguminosae, and Poaceae had the same directions and similar magnitudes among or between different families (Figure 8b).

**Figure 9:**
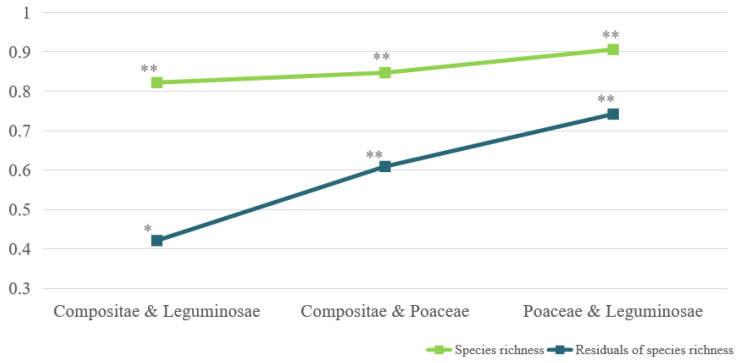
Correlation coefficients of IAP species richness of Compositae, Leguminosae, and Gramineae families before and after controlling for common environmental and anthropogenic factors. * *p* < 0.05, significant at 0.05 level; ** *p* < 0.01, significant at 0.01 level.

Correlation analysis of the species richness of Compositae, Leguminosae, Poaceae, Amaranthaceae, Solanaceae, Onagraceae, Euphorbiaceae, and Convolvulaceae in various provinces showed that the correlation coefficients between the majority of families exceeded 0.7, and the correlation coefficients between Compositae and the other families were all above 0.7. The correlation coefficients between Compositae and the families of Leguminosae, Poaceae, Solanaceae, Euphorbiaceae, and Convolvulaceae exceeded 0.8. Taking the Asteraceae, Leguminosae, and Poaceae families as examples, after controlling for the influence of common environmental and anthropogenic factors, the correlation coefficients of the residuals of IAP species richness decreased. The correlation coefficient between Poaceae and Leguminosae decreased from 0.906 (*p* < 0.01) to 0.742 (*p* < 0.01), while the correlation coefficient between Compositae and Poaceae decreased from 0.847 (*p* < 0.01) to 0.609 (*p* < 0.01). The correlation coefficient between Compositae and Leguminosae decreased the most and ranged from 0.822 (*p* < 0.01) to 0.421 (*p* < 0.05) (Figure 9).

## 3. Discussion

### 3.1. Species Composition of IAPs in China

IAPs have caused massive ecological disasters and economic losses in China [39]. The most harmful invasive plants in China include *Alternanthera philoxeroides*, *Eichhornia crassipes*, and *Ageratina adenophora* [34]. The results of the present study showed that at the family level, Compositae, Leguminosae, Poaceae, and Amaranthaceae accounted for the largest proportion of IAPs in China. This finding is similar to global research indicating that Compositae (1343 species), Poaceae (1267 species), and Leguminosae (1189 species) account for the largest proportion of IAPs worldwide [40]. According to the intrinsic superiority hypothesis (ISH), exotic species can successfully invade new sites due to some of their inherent characteristics, including morphological, physiological, ecological, genetic, and behavioral characteristics, which enable them to perform better than native species in resource acquisition, habitat adaptation, or population expansion [39]. For example, studies have shown that Asteraceae plants generally have allelopathic effects, making them often aggressive towards neighboring plants and leading to the formation of a single community of Asteraceae plants [41]. The findings of the present study revealed differences in the life forms of IAPs from different families. The IAPs from Compositae, Amaranthaceae, Poaceae, and Solanaceae were mainly annual or biennial, while those from Leguminosae were mainly perennial herbs, shrubs, or trees. Different life forms of plants differ in invasiveness. Plants with shorter life histories, such as annual or biennial IAPs, have advantages in the introduction and colonization stages, while perennial IAPs have advantages in the expansion and invasion stages [42].

### 3.2. IAP Distribution Patterns

The management system of invasive alien species (IAS) consists of early-warning, monitoring, eradication, and spread-blocking technologies [18]. Revealing the species-distribution patterns through models is one of the common tools for preventing, managing, and controlling IAS [18]. This study comprehensively analyzed the distribution patterns of IAPs in China. The synthetic scores indicated that provinces including Jiangsu, Guangdong, Fujian, Taiwan, Hebei, Shandong, Yunnan, Anhui, and Zhejiang showed higher risks of IAP invasion. In addition, the first records of provinces Guangdong, Taiwan, and Yunnan were relatively high and were under high risk of the introduction of IAPs, and it is urgent to strengthen inspection and quarantine at the entrances in these provinces. High invasion risk might be due to the natural conditions in the eastern coastal provinces and Yunnan, which provide IAPs with more suitable habitats for colonization. At the same time, frequent socio-economic activities in these provinces, including tourism and international trade, have also increased the propagule pressure of IAPs and accelerated the introduction and dispersal of IAPs. Some provinces, namely Hubei, Sichuan, Shaanxi, and Anhui, had high species similarity coefficients. It is inferred that these mid-latitude provinces play a greater role in the dispersal of IAPs and pose high risk of invasion.

There were strong correlations between various families of IAPs. Regions with high species richness of IAPs of one family generally had higher species richness of other families, indicating that these regions had higher species richness of IAPs due to the joint invasion of multiple species from multiple families, rather than a single family or a small number of families. A similar finding showed that there was a high congruence in the distribution of invasive alien animals and IAPs in China [31,43]. The present study also indicated the importance of common environmental and anthropogenic factors to the spatial distribution congruence of IAPs. Especially between the Compositae and Leguminosae, the positive correlation became much weaker after removing environmental and anthropogenic factors. Nevertheless, there was still a significant positive correlation (*r* = 0.421, *p* < 0.05). That is, environmental and anthropogenic factors could not fully explain the spatial distribution congruence of IAPs in different families, especially in the Leguminosae and Poaceae families and in the Poaceae and Compositae families. It was inferred that other factors, such as the mutually beneficial synergistic invasion effect between plants, as pointed out by the invasional meltdown hypothesis (IMH), led to the spatial distribution congruence of IAPs in different families [44,45]. It has been shown that when invasive plants are nitrogen-fixing plants, the available nitrogen content in the invaded area is higher, and soil with high nitrogen content is more conducive to the colonization or dispersal of other invasive plants [46]. Invasive woody plants often create more favorable habitats for other invasive shrubs or herbaceous plants [47]. However, further studies are required to confirm whether this collaborative invasion mechanism still occurs on a larger scale.

One of the most well-recognized species-distribution patterns in nature is that the similarity of species composition is lower when regions are farther apart, which is also the result of environmental filtering [48]. The distribution of IAPs in China was also in line with this pattern, and provinces with high similarity coefficients of IAP species composition were often geographically adjacent provinces, such as Jilin and Heilongjiang and Gansu and Qinghai. The species similarity coefficients of provinces in the middle latitude were higher than those at higher or lower latitudes. Although IAPs have strong environmental adaptability, their distribution is still influenced and constrained by environmental factors. The average similarity coefficients of Hainan and Taiwan were low, which might be due to the limitations of island attributes and the tropical and subtropical climates of these two provinces on the dispersal of IAPs to high latitudes. Nevertheless, with the global rise in temperatures and the development of trade and tourism, IAPs are likely to continue to break through biogeographical barriers and disperse between islands and continents [49].

### 3.3. Influences of Multiple Factors on the Spatial Distribution of IAPs of Different Families

Previous studies have attempted to clarify the major influencing factors of the distribution pattern of IAPs in China [31]. Some studies have reported that biogeography and socio-economic factors are both important [33,50], while some studies have concluded that environmental factors play a more important role [15,32,51]. Generally, for native plants, low temperature is the primary limiting factor affecting distribution [40]. This study showed that, for species richness of IAPs, more environmental factors with VIP values higher than 1 were retained in the optimal model compared to anthropogenic factors. The prediction power of PET, precipitation, and temperature was better, reflecting that the distribution of IAPs was also constrained by the environment, which was consistent with the conclusion of the IAP species similarity coefficient analysis. Visitors were the most important predictor for the first records. Compared to species richness, the first records of IAPs were difficult to predict and were influenced by anthropogenic factors. Previous studies have shown that most IAPs have been unintentionally or intentionally introduced into China, and only a small number have been introduced through natural dispersal [52]. There are various carriers of unintentional introduction, and IAPs, including *Pseudosorghum fasciculare*, *Solanum rostratum*, and *Lolium temulentum*, were mixed in imported grain and unintentionally introduced into China, while *Praxelis clematidea* was unintentionally introduced along with ornamental plants. *Symphyotrichum subulatum* and Plantago virginica were unintentionally introduced to China through agricultural trade or travel [52]. Therefore, compared to the distribution pattern of IAP species richness, there might be great uncertainty in the first records of IAPs. Based on the results of this study, it could also be inferred that anthropogenic factors played more important roles in the IAP introduction stage than in the dispersal stage. The prevention and control of biological invasions is challenging because once the invasive alien species break out in a new area, it becomes difficult or almost impossible to eradicate them [11,53]. Therefore, early detection and rapid response are crucial to prevent and control the outbreak of new biological invasions [11]. Currently, the developing and emerging economies are in a stage of rapid development, while climate warming and extreme temperatures have led to frequent changes in the natural environment. It has been found that IAPs can benefit more from global elevated temperatures and CO_2_ enrichment than native plants [54]. With the combined impact of environmental and anthropogenic factors on the distribution pattern of IAPs, these changes will undoubtedly exacerbate the threat of biological invasion [38,55].

## 4. Materials and Methods

### 4.1. Dataset

In this study, the IAPs referred to IAPs that entered China from abroad. The data were obtained from China’s Invasive Alien Species (revised edition) [52], which is an updated inventory of IAPs that have established populations in China. The primary data were derived from papers in peer-reviewed journals, books, databases, webpages, and field surveys on IAPs in China [52]. The collected information included the classification status, distribution range, introduction pathway, first detection time and sites, origin, habitat type, and life history of IAPs. Furthermore, referring to Ma and Li [36], according to the invasion characteristics of IAPs and the magnitude of damage caused by invasion, IAPs were divided into five levels. Level 1 consists of malignant IAPs that have caused great economic losses and serious ecological impacts at the national level and invaded more than one province-level administrative region. Level 2 consists of serious IAPs that have caused great economic losses and ecological impacts at the national level and invaded at least one province-level administrative region. Level 3 consists of local IAPs that are distributed in more than one province-level administrative region and have only caused local damage. Level 4 contains IAPs that have been inferred not to cause significant ecological impacts or economic losses. Level 5 refers to IAPs that need to be observed, and mainly includes newly emerged or reported IAPs for which relevant reports are still insufficient to determine their future trends. In this paper, province-level administrative regions were used as the basic geographical units for analysis (including 23 provinces, five autonomous regions, four municipalities, and two special administrative regions). Four municipalities (Beijing, Tianjin, Shanghai, and Chongqing), as well as two special administrative regions (Hong Kong and Macao), were merged into their neighboring provinces; that is, Beijing and Tianjin were merged into Hebei, Shanghai was merged into Jiangsu, Chongqing was merged into Sichuan, and Hong Kong and Macao were merged into Guangdong (Figure 1).

### 4.2. Invasion Indices

This study used five indices to evaluate the magnitude of invasion in each province: the species richness of IAPs, the first records of IAPs, the average similarity coefficient of IAPs, the average risk score of IAPs, and the relative species richness of IAPs.

The average similarity coefficient of IAPs for different regions were calculated based on Jaccard’s similarity coefficient [56], using the following formula:

(1) $$SC_{i}=\frac{\sum_{j=1}^{27}\frac{c_{ij}}{a_{i}+b_{j}-c_{ij}}}{27}.$$

In this formula, *SC_i_* is the average Jaccard’s similarity coefficient of the *i*-th province, *c_ij_* is the species richness shared between regions *i* and *j*, and *a_i_* and *b_j_* are the total species richness in regions *i* and *j*, respectively.

The average risk score of IAPs was calculated using the following formulas:

(2) $$RS_{i}=\frac{\sum_{m=1}^{p_{i}}rs_{m}}{p_{i}},$$

(3) $$rs_{m}=6-rr_{m}.$$

In these formulas, *RS_i_* is the average risk score of the *i*-th province. *rr_m_* is the risk rank for the *m*-th species, *rs_m_* is the risk score for the *m*-th species, and *p*_i_ is the IAP species richness of the *i*-th province.

The relative species richness of IAPs represented the ratio of the IAP species richness to the native plant species richness, reflecting the proportion of IAPs in native communities. The native plant species richness data were obtained from http://www.sp2000.org.cn (accessed on 10 January 2023).

### 4.3. Environmental and Anthropogenic Factors

Previous studies showed that the combination of environmental and anthropogenic factor variables can effectively predict the distribution pattern of invasive alien plants [15,31,32,33,37,50]. For example, Li and Shen selected seven climatic variables and three socio-economic variables to analyze their role in shaping the distribution patterns of IAPs [15]. Zhou et al. evaluated the impact of six climatic factors and three socio-economic variables on the differential strategies of IAPs [50]. We integrated previous studies to select variables that are assumed to affect the distribution pattern of IAPs. Finally, seven environmental factor variables were collected: (1) temperature; (2) precipitation; (3) temperature annual range (hereinafter temperature range); (4) potential evapotranspiration (PET); (5) actual evapotranspiration (AET); (6) latitude; and (7) area. Five anthropogenic factors were collected: (8) the number of visitors (hereinafter visitors); (9) transportation volume; (10) population; (11) gross domestic product (GDP); and (12) human footprint.

Temperature, precipitation, and temperature range data were downloaded from the worldclim database (http://www.worldclim.org) (accessed on 25 January 2023). PET and AET were obtained from Qian [57]. Visitors, transportation volume, population, and GDP data were collected from the China Statistical Yearbook 2019 (http://www.stats.gov.cn/sj/ndsj/2019/indexch.htm) (accessed on 27 January 2023). The human footprint was obtained from Venter et al. [58].

### 4.4. Statistical Analysis

Ordinal least-squares (OLS) regressions were used to analyze the distribution patterns of various IAP invasion indices along the latitude or longitude. The first records were log-transformed to improve their normality. Principal component analysis (PCA) was used to extract the principal components of the five invasion indices of IAPs, and principal component coefficients based on the factor loading matrix were obtained. Then, the scores of each principal component were calculated to aggregate the synthetic scores of invasion risks for each province [59]. The entropy method was used to weight each index based on the information it contained [60,61].

To avoid collinearity between explanatory variables, the partial least-squares (PLS) regression method was used to establish regression models of environmental and anthropogenic factors on the species richness and first records of IAPs. In the PLS regression model, the variable importance for the project (VIP) value was used to quantify the importance of each independent variable in the model [62]. Standardized regression coefficients were used to quantify the explanatory power and direction of each predictor on the response variable [63]. The cross-validation method was used to determine the components that should be retained in the optimal model. *Q*^2^ represented the percentage of variation predicted by a component through cross-validation, and *Q*^2^_cum_ represented the percentage of variation predicted by all retained components. The larger the *Q*^2^_cum_ (*Q*^2^_cum_ > 0.5), the better the prediction ability of the model [64]. The selection of variables and the process of obtaining the optimal model referred to Shi et al. [64].

The PLS regression method was further used to establish regression models of environmental and anthropogenic variables on the species richness of IAPs of Compositae, Leguminosae, and Poaceae. The correlation analysis showed high positive correlations between the species richness of the IAPs of Compositae, Leguminosae, and Poaceae. Considering the common environmental and anthropogenic factors affecting the distribution patterns of IAPs of different families, the residuals were then extracted from the PLS regression models. The residuals were used as surrogates of the IAP species richness of each family, the correlations of the residuals of IAP species richness were calculated, and the impact of common external factors on the spatial distribution congruence of different families was evaluated.

OLS regression analysis and PCA were performed using R software, while PLS regression analysis was performed using SIMCA 14.0.

## 5. Conclusions

This study comprehensively analyzed the distribution patterns of IAPs in China. The first records of provinces Guangdong, Taiwan, and Yunnan were relatively high and were at high risk of the introduction of IAPs. It is urgent to pay attention to the introduction of alien species and strengthen inspection and quarantine at the entrances in these provinces. The provinces of Hubei, Sichuan, Shaanxi, and Anhui had high species similarity coefficients, and the dispersal of IAPs in these provinces should be particularly controlled. The synthetic scores indicated that the provinces of Jiangsu, Guangdong, Fujian, Taiwan, Hebei, Shandong, Yunnan, Anhui, and Zhejiang showed higher risks of IAP invasion. For species richness of IAPs, more environmental factors with VIP values higher than 1 were retained in the optimal model, reflecting the importance of environmental filtering on IAPs. Visitors were the most important predictor for first records of IAPs. Compared to species richness, first records were difficult to predict and were influenced by anthropogenic factors. There was spatial distribution congruence of various families of IAPs, and the external factors could not fully explain the spatial distribution congruence. The prevention and control of IAPs also needs to consider the interactions of multiple IAPs within the region, breaking the synergy between IAPs. The findings of this study could enrich the relevant research on IAP invasion mechanisms and provide suggestions for regional IAP prevention and control.

## Data Availability

Not applicable.
